# Striving for population-level conservation: integrating physiology across the biological hierarchy

**DOI:** 10.1093/conphys/coaa019

**Published:** 2020-04-04

**Authors:** Elizabeth M Ames, Meaghan R Gade, Chelsey L Nieman, James R Wright, Christopher M Tonra, Cynthia M Marroquin, Annalee M Tutterow, Suzanne M Gray

**Affiliations:** 1 School of the Environment and Natural Resources, The Ohio State University, 2021 Coffey Road, Columbus, OH 43210, USA; 2 Departmant of Evolution, Ecology and Organismal Biology, The Ohio State University, 318 W. 12th Ave., Columbus, OH 43210, USA

**Keywords:** Biological Hierarchy, conservation, physiology, scaling up

## Abstract

The field of conservation physiology strives to achieve conservation goals by revealing physiological mechanisms that drive population declines in the face of human-induced rapid environmental change (HIREC) and has informed many successful conservation actions. However, many studies still struggle to explicitly link individual physiological measures to impacts across the biological hierarchy (to population and ecosystem levels) and instead rely on a ‘black box’ of assumptions to scale up results for conservation implications. Here, we highlight some examples of studies that were successful in scaling beyond the individual level, including two case studies of well-researched species, and using other studies we highlight challenges and future opportunities to increase the impact of research by scaling up the biological hierarchy. We first examine studies that use individual physiological measures to scale up to population-level impacts and discuss several emerging fields that have made significant steps toward addressing the gap between individual-based and demographic studies, such as macrophysiology and landscape physiology. Next, we examine how future studies can scale from population or species-level to community- and ecosystem-level impacts and discuss avenues of research that can lead to conservation implications at the ecosystem level, such as abiotic gradients and interspecific interactions. In the process, we review methods that researchers can use to make links across the biological hierarchy, including crossing disciplinary boundaries, collaboration and data sharing, spatial modelling and incorporating multiple markers (e.g. physiological, behavioural or demographic) into their research. We recommend future studies incorporating tools that consider the diversity of ‘landscapes’ experienced by animals at higher levels of the biological hierarchy, will make more effective contributions to conservation and management decisions.

## Introduction

**Figure 1:**
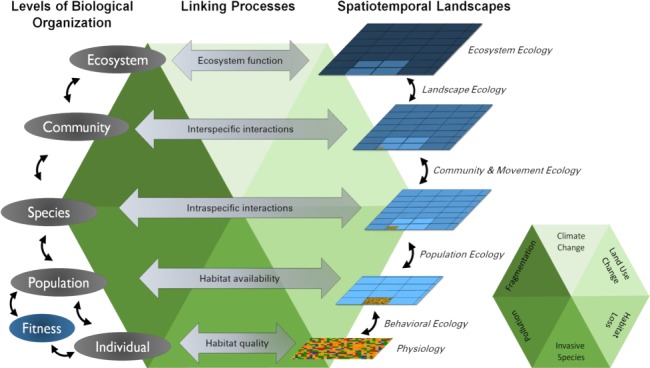
A conceptual diagram of the complexities of scaling up the biological hierarchy from individuals through ecosystems. A major aim of conservation physiology is to provide mechanistic links between physiological responses to human-induced environmental change (underlying green hexagon; key inset, bottom right) and population declines, and further to the ecosystem level response. Each biological level in the hierarchy (far left) occurs at increasingly broader spatial and temporal scales (blue boxes; Spatiotemporal Landscapes) and are linked to each other through various biological processes (examples given in grey arrows; Linking Processes). The green hexagon represents major human-induced environmental stressors that are known, or are expected, to influence these processes. For example, if habitat quality is degraded such that temperature extremes are common throughout the habitat, then individuals may shift patterns of behavioural thermoregulation by choosing new habitats that optimize their performance locally. Scaling up to the population level, though, requires information about the availability of preferred habitat and how that might be altered, for example by intraspecific interactions and how this affects reproductive output (i.e. fitness). As we strive to understand how individual-level responses to altered environments scale up to species, integration of not just how animals use the fine scale habitat but how they move throughout the entire landscape (e.g. movement ecology) is critical. Each step-up in the hierarchy results in a more complex abiotic and biotic landscape that influences the processes guiding interactions among individuals and species. For example, at the community level, the landscape represents layers of individual-level habitat choice that are dictated by physiological state, interactions among conspecifics and interspecific (e.g. predator-prey) dynamics. Finally, at the ecosystem level, we expect the entire suite of interactions to be reflected in the way that an ecosystem functions—or does not function—depending on the scope of environmental change. Finding these links and scaling up will require collaboration and integration across disciplines (examples given in italics, far right).

Through the explicit incorporation of physiology into conservation science, the field of conservation physiology (formally introduced by Wikelski and Cooke, 2006) has provided novel insights into the physiological processes underlying population declines, biodiversity loss and other ecological patterns in the face of human-induced rapid environmental change (HIREC; Sih *et al*., 2011; Table 1). Conservation physiology aims to be an integrative scientific discipline that unveils cause-and-effect relationships from the individual to the population by exploring the mechanisms of response to stressors and environmental change. Further, by leveraging techniques from other disciplines such as genomics, immunology, landscape ecology and sensory physiology, conservation physiology spans multiple levels of the biological hierarchy (Madliger *et al*., 2016). While there have been many successes towards conservation physiology goals (see Madliger *et al*., 2016; Bergman *et al*., 2019), we suggest that the field is still in need of making explicit links across the levels of the biological hierarchy (i.e. individual, population, species, community, ecosystem; Fig. 1) to better reveal populations in jeopardy and inform conservation and management strategies.

**Table 1:** Resources for integrating *Conservation Physiology* with other disciplines

**Disciplinary link to Conservation Physiology**	**Examples of pertinent literature**	**Description**
*Definitions and refinements of Conservation Physiology*	Carey, 2005	Integration of physiological methods with conservation biology and using these links to inform cause-and-effect relationships amid anthropogenic environmental change.
	Stevenson *et al*., 2005	Synopsis of ways in which ecotoxicology and conservation biology can converge using common physiological approaches, introduce the term conservation physiology.
	Tracy *et al*., 2006	Placement of physiological ecology at the base of conservation as means for understanding mechanisms of response to anthropogenic environmental change.
	Wikelski and Cooke, 2006	First formal definition of Conservation Physiology.
	Metcalfe *et al*., 2012	Revision of Conservation Physiology definition as an applied subdiscipline of ecophysiology (marine fish example).
	Seebacher and Franklin, 2012	Revision of Conservation Physiology definition with a focus on human impacts on the environment and conceptualized mechanistic approach.
	Cooke *et al*., 2013	Refinement of original *Conservation Physiology* definition to more broadly include all taxa and conservation and management strategies (e.g. restoration initiatives) provides extensive list of sub-disciplines with conservation physiology links.
	Coristine *et al*., 2014	Placement of physiological ecology at the base of conservation as means for understanding mechanisms of response to anthropogenic environmental change.
	Lennox and Cooke, 2014	Analysis of literature that seeks to integrate conservation and physiology.
	Madliger and Love, 2015	Synopsis of ways that physiological tools can be used to integrate over multiple disciplines, not just conservation biology.
	Madliger *et al*., 2016	Review of successful conservation physiology studies (i.e. projects successfully use physiological principles to inform conservation initiatives, human behaviour changes and policy.
	Cooke *et al*., 2017	Evaluation of progress of Conservation Physiology as a field.
	Madliger *et al*., 2017	Perspective on how conservation physiology can, as a field, support a positive outcome for understanding how the natural world responds to anthropogenic environmental change.
	Madliger *et al*., 2018	Synthetic review of physiological approaches, or ‘tools’, that can effectively be used for bridging disciplines and advancing the field of conservation physiology.
	Tomlinson *et al*., 2018	Review of ways in which conservation physiology borrows tools (crosses boundaries) from across disciplines to enhance our ability to detect individual-level responses and apply it to conservation of declining populations.
*Stress and Energetics*	Beaulieu *et al*., 2013	Perspective on using oxidative stress markers as links between individual fitness and population demographics.
	Beaulieu and Constantini, 2014	Review of utility of specifically incorporating the use of oxidative stress markers to scale from individuals to populations in conservation physiology studies.
	Dantzer *et al*., 2014	Review and meta-analysis linking stress responses (e.g. glucocorticoid levels) with human disturbance of the environment in a conservation physiology context.
*Nutritional Ecology*	Raubenheimer *et al*., 2012	Review on the temporal relationships between nutritional ecology and conservation physiology.
	Birnie-Gauvin *et al*., 2017	Review on the effects of rapid environmental change on animal diets.
*Behavioral Ecology*	Killen *et al*., 2013	Perspective paper assessing the influence of environmental stressors on correlations between behaviour and physiology.
	Cooke *et al*., 2014	Perspective on the need to integrate the disciplines of physiological and behavioural ecology to better meet the aims of conservation physiology.
*Habitat Quality*	Homyack, 2010	Review of physiological metrics that could inform population vital rates and habitat quality, thus providing needed links for conservation goals.
	Albano, 2013	Perspective on the need to integrate physiology into assessment of habitat quality in studies of birds.
*Landscape Ecology*	Ellis *et al*., 2012	Review of the integration of physiological information with spatial information can lead to a better understanding of landscape effects on population persistence
	Ziv and Davidowitz, 2019	Conceptual framework for integrating landscape ecology (heterogeneous/fragmented landscapes) and physiology (life history/reproductive output)
*Macrophysiology*	Gaston, 2009	Perspective on how variation in population parameters can determine range shifts.
	Chown and Gaston, 2008	Perspective on the ability of macrophysiology, combined with physiology, to understand threats to biodiversity.
	Chown and Gaston, 2016	Review of the applications and challenges facing macrophysiology.
*Scaling up*	du Toit, 2010	Review of studies that include considerations of scale in solving conservation problems.
	Cooke *et al*., 2014	Review of conservation literature from marine systems that integrate scale, including examples of scale of policy and policy application.
	Bergman *et al*., 2019	Review of case studies that have successfully scaled from individual-level physiological measures to population-level conservation.
*Climate Change Ecology*	Pörtner and Farrell, 2008	Perspective on how animal responses to increased temperature are largely physiologically based in basis and understanding physiology can reveal key insights into future ecological trends.
	Helmuth, 2009	Perspective on the need for integrated, system-based approaches to predicting animal responses to changing climate, as responses are likely to be driven by physiology and not by simple abiotic gradients.
	Bozinovic and Pörtner, 2015	Perspective on the need to incorporate physiology to understand the full effects of climate change on ecology and evolutionary biology.
	Evans *et al*., 2015	Perspective on the need to incorporate physiology into mechanistic models to understand range distributions in a changing climate.
*Restoration Ecology*	Cooke and Suski, 2008	Perspective on benefits of integrating physiology with restoration efforts.
	Tarszisz *et al*., 2014	Review and integration of physiological metrics in translocation studies for restoration of rare and endangered species.
*Migration*	Jachowski and Singh, 2015	Review of recent advances in understanding of physiology can inform studies on animal movement.
	Lennox *et al*., 2016	Perspective on the use of physiological and behavioural mechanisms to understand animal migration and interactions with anthropogenic threats.
*Management*	Cooke and O’Connor, 2010	Perspective on the challenges in adopting management strategies based on conservation physiology.
	Madliger, 2012	Perspective on the use of sensory ecology to inform conservation and management.
	Dick *et al.*, 2016	Perspective on the need to adopt multidisciplinary approaches that bridge disciplines as well as engage policy makers and other stakeholders in conservation.
	Mahoney *et al*., 2018	Review of the use of physiology to inform species recovery plans, revealing a disconnect between conservation physiology and their use in development of management plans.

A non-exhaustive list of papers that define and refine conservation physiology for practitioners interested in starting to incorporate conservation physiology in their own research or management practices. We also include references, organized by discipline or specific topic of interest, that provide useful information or examples for combining conservation physiology with other fields to facilitate crossing disciplinary boundaries and scaling up the biological hierarchy

Most often, conservation physiology studies collect physiological measures at the individual level, with limited ability to directly scale results to population responses. Studies often rely on a ‘black box’ to make population-level predictions, without explicit links between physiological responses to the environment (e.g. hypothalamic–pituitary–gonadal axis) and population vital rates (e.g. survival, recruitment). Such individual-level data can be inherently problematic for conservation physiology as most conservation action needs to be implemented at the population-level or higher to produce meaningful results (Cooke and O’Connor, 2010). Despite considerable attention over the last 30 years, the concept of scale and the implementation of ‘scaling up’ the biological hierarchy has remained a challenge for ecologists (Scholes, 2017). Scale, in relation to ecological research, is most commonly defined as the extent in space and the duration in time of a phenomenon or object of study (O’Neill *et al*., 1986). We use the term *scaling up* to refer to moving from a lower to higher level of the biological hierarchy to make broader-scale inferences, with the goal of strengthening conservation decisions and management practices.

Measurements of response made at the individual level can be scaled to population inference by demonstrating that changes in physiological responses impact vital rates and fitness, which subsequently are expected to impact population dynamics. In Fig. 1, we outline possible links between habitat and landscape features impacted by HIREC and the level of the hierarchy expected to be most directly affected. Here, we refer to *habitat* as the immediate abiotic and biotic environment experienced by an individual, and *landscape* refers to a collection of habitats used by a population or species (Fig. 1). As a hypothetical example, habitat quality could be disrupted due to changes in the thermal landscape when trees are removed from a field. We might expect an individual, say an ectothermic lizard, to make behavioural choices to mitigate the effects of variable temperature on metabolic processes (physiology). The choice to move may have unintended consequences associated with acquisition of resources (e.g. shade) and interactions with other individuals (behavioural ecology), thus affecting reproductive success and individual fitness that translates to population declines (population ecology). This example highlights that understanding just the physiology, behaviour or population demography of a species, as opposed to all three, may not be enough for long-term conservation goals. Carrying our example further, understanding the thermal needs of an individual lizard and how individuals interact across the landscape could inform placement of trees in habitat restoration initiatives. Achieving effective population (and beyond) inference that leads to actionable conservation requires a collaborative, multidisciplinary approach to create the explicit links across the biological hierarchy (Fig. 1). Bridging conservation physiology with other ecological fields provides a valuable opportunity to generate powerful insights that span the ‘knowledge-action gap’ for conservation and management (Cooke, 2018).

Here, we outline a number of examples where conservation physiology has been applied, both successfully and less effectively, to scale from individual- to population-level inference, population to species-level inference and populations or species to higher levels of the biological hierarchy, to ultimately inform conservation recommendations. While this is not an exhaustive review, we attempt to highlight examples that effectively use interdisciplinary research to make connections across the biological hierarchy, as well as studies that incorporate physiological measures and serve as potential opportunities for broader scale inferences. We argue that investigation across the levels of biological hierarchy, most effectively accomplished by crossing disciplinary boundaries (Dick *et al*., 2016), is necessary to open the ‘black box’ and facilitate conservation success. Ultimately, we hope to provide guidance on how conservation physiology, in conjunction with other disciplines, can be leveraged to scale beyond individual measures to provide robust conservation and management decisions.

## Scaling up the biological hierarchy

### From individuals to population and species

Recent advances in technology, computational ability, empirical models and interdisciplinary approaches have increased our ability to scale up from individual measurements to population and species level inference (see Table 2). In turn, a number of reviews and perspectives have emerged that encourage the integration of conservation physiology with other disciplines to better address conservation needs and the role of physiology in achieving key conservation goals (Table 1). These authors emphasize the importance of incorporating physiological metrics, measured at the individual level, with other disciplines to gain a mechanistic understanding of how processes associated with population declines function (e.g. see Madliger *et al.*, 2018). While there has been a push for incorporating physiology into other ecological disciplines, there has been less emphasis on simultaneously investigating links among physiological traits and their interactive effects on population responses to environmental change. A recent review by Bergman *et al*. (2019) outlines several case studies that successfully integrate environmental change, physiology and demographic measures to scale from the individual to population-level conservation action. Incorporating environmental, physiological and demographic metrics offers a useful conservation tool; however, many stressors studied at the individual level are likely impacted by a suite of factors requiring integration of metrics and disciplines beyond the three listed. An exemplary case study involves the well-known threespine stickleback (*Gasterosteus aculeatus*). Our knowledge of this species, from genomic architecture, sensory physiology, individual behaviours, population dynamics in natural and disturbed environments, and speciation, could potentially yield a suite of possible links among levels of the biological hierarchy; however, the mechanistic links between these dynamics and applied conservation have yet to be elucidated (see Box 1). In this section, we provide examples where two or more individual-level measures can be combined to enhance our understanding of the link to populations and species.

**Table 2:** Tools for scaling up the biological hierarchy

**Scaling level**	**Tools and approaches**	**Description**	**Examples/references**
Individual to population	Multiple physiological markers	Sampling a suite of physiological markers from individuals can strengthen interpretations of fitness consequences or lead to discoveries of unanticipated fitness consequences of stressors.	Burgess *et al*., 2017; Madliger *et al*., 2018,
Individual to population	Spatial modelling	When used carefully while accounting for greater heterogeneity at larger scales, various spatial modelling techniques can allow small-scale studies to make inferences at larger spatial scales.	Keitt and Urban, 2005; Evans *et al*., 2015; Scholes, 2017
Individual to population to species	Data sharing	Online data repositories of individual physiological measurements, or population demographic metrics, can help researchers spatially or temporally expand their study. Particularly important for meta-analyses, or when research is not published.	Dryad [https://datadryad.org/], GenBank [https://www.ncbi.nlm.nih.gov/genbank/], Movebank [https://www.movebank.org/],
Individual to population to species	Bridging disciplines	Crossing disciplinary boundaries, through interdisciplinary collaborations or incorporating measures outside of one’s area of expertise (e.g. physiologists incorporating behavioural observations and vice versa), can ultimately link changes in population demographics to physiological effects, or elucidate physiological mechanisms for observed population changes.	See Table 1
Individual to population to species to Community	Agent-Based Modelling	ABM can be used to integrate individual-level behaviours and physiological response with multiple environmental conditions (abiotic and social) and may therefore prove useful in combining multiple landscapes at multiple scales.	McLane *et al*., 2011
Individual to population to species to Ecosystem	Macrophysiology	Investigating physiological variation across large spatial, temporal or phylogenetic scales (Chown and Gaston 2008), can shed light on how large-scale stressors (e.g. habitat fragmentation and degradation, climate change) influence populations, particularly when coupled with demographic measures.	Chown and Gaston, 2008; Lea *et al*., 2018
Individual to population to species to ecosystem	Collaboration	Large networks of organizations, or smaller working groups of researchers, are an effective means of increasing the spatial scale of data collection and conservation inferences.	Partners in Flight; Partners in Amphibian and Reptile Conservation; Blanding’s Turtle WG; Prothonotary Warbler WG; Rusty Blackbird WG; Bat Conservation International; North American Bat Monitoring Program; MarAlliance; Southern Ocean Research Partnership; Urban Wildlife Information Network; Migratory Dragonfly Partnership; Pacific Northwest Native Freshwater Mussel WG

### Behavioural and sensory ecology

An understanding of sensory ecology is a key factor in evaluating responses to HIREC as it provides information about the ability of animals to detect change (Wikelski and Cooke, 2006; Sih *et al*., 2011; Madliger, 2012). Further, behavioural adjustments are often considered the first observable response to an altered environment (Sih *et al.,* 2011); thus, combining work from these two fields can inform how we expect individuals to respond, as well as potential repercussions for populations. As an example, mate choice and reproductive isolation in many Lake Victoria haplochromine cichlid fishes depends on female visual sensitivity and their perception of male nuptial coloration. Cultural eutrophication and elevated turbidity, however, has resulted in narrowing of the visual spectrum of light and altering sexual selection dynamics, ultimately contributing to reduced species diversity (Seehausen *et al*., 1997; Seehausen *et al*., 2008). Many sensory-based studies do not explicitly link between the physiological effect of a stressor on an individual and measurable effects on population dynamics. For example, Martín and López (2013) found a decrease in the efficacy of mountain lizard (*Iberolacerta cyreni*) scent marks under increased temperatures, thereby reducing the ability of females to find chemosensory cues that play a significant role in sexual selection. This study successfully demonstrates the link between a globally important stressor (elevated temperature) and sensory and behavioural responses. Future studies wishing to scale up to the population level would need to explicitly measure population level consequences. For example, by implementing landscape ecology spatial modelling (Table 2) that incorporates thermal habitats, chemosensory cue degradation and population size (reproductive output) we may be able to elucidate broader population-level conservation impacts.

Cooke *et al*. (2013) emphasize the merit of integrating behavioural ecology and conservation physiology to successfully advance conservation goals. In many instances, behavioural responses to individual physiological stressors can provide a link between human-induced stressors and higher levels of the biological hierarchy (Box 2). Conservation strategies employing behavioural information have been used to attract or repel animals from particular areas (e.g. reduction of human–wildlife conflicts) and for the improvement of captive breeding programs (Busch and Hayward, 2009; Cooke *et al*., 2013; Madliger *et al*., 2016). Additionally, behavioural changes can provide insight into potential effects, or even presence of, stressors. An understanding of how different stressors, such as chemical pollutants, are likely to interact with and alter behaviour may provide researchers with clues about how stressors impact population-level and subsequently species-level processes.

Understanding individual-level physiology can provide key baseline information, that when scaled appropriately, may provide insight into higher scale responses to environmental change. For example, walleye (*Sander vitreus*) are negatively phototaxic and require cold habitat. By linking physiologically determined thermal tolerance and visual sensory requirements, managers can determine critical habitat areas such as the space available in the water column in which walleye can forage (Lester *et al*., 2004). Determination of critical habitat can in turn aid fisheries managers by indicating areas in which fishing may be more or less productive or should be of conservation concern for this species. Further, agent-based modelling could provide insight into the specific population-level effects of individual physiology and multiple environmental stressors (reviewed in McLane *et al*., 2011; Table 2) allowing for the scaling of these physiological mechanisms across levels of the biological hierarchy.

### Population and movement ecology

Population responses (e.g., growth, decline, recruitment, age/sex ratio) to environmental stressors are often determined by long-term demographic studies. While there is value in using population demography to make conservation determinations for species, or inferring habitat quality in some cases (Johnson, 2007), such traditional approaches may lack immediacy in identifying a conservation problem, directly linking environmental stressors to population condition or insight into conservation strategies. In their review, Ellis *et al.* (2012) highlight the importance of blending physiology with landscape ecology (coining the term ‘landscape physiology’) as a way to provide early detection of declining populations in altered habitats. They further suggest that physiological measures (e.g. glucocorticoids) can provide direct quantification of population condition and therefore demonstrate expected, immediate population responses to landscape change. For example, Cosgrove *et al*. (2017) assess chronic stress in eastern yellow robins (*Eopsaltria australis*; as measured by heterophil: lymphocyte ratios), while also characterizing vegetative structure to reveal habitat and nutritional covariates related to potential population declines. While this study does not show that heterophil:lymphocyte ratios predicted local population extirpations, the research design is well-suited to scaling up from individual to population-level effects.

Landscape physiology offers a new and potentially powerful approach that allows for broader application of certain data by integrating behaviour and physiology that are shaped by environmental and landscape features. However, we argue that without the integration of other disciplines (e.g. population ecology, urban ecology, ecotoxicology) with landscape physiology, limitations still exist for population-level inference. Population-level responses to changes in habitat quality still elude many studies but can ultimately be reached by increasing spatial scope and integrating other disciplines. For instance, in a longitudinal study on ringed seal (*Pusa hispida*) population demographics, Ferguson *et al.* (2017) integrated elements of reproductive biology, stress physiology and population ecology to link large-scale sea ice dynamics to ringed seal population declines. They found that increased open water periods resulted in decreased body condition and higher stress levels over a period of 18 years. In addition, an extreme open water year coincided with high stress levels and low ovulation and pregnancy rates, leading to subsequent declines in pup recruitment.

Other studies have attempted to make population-level inferences by incorporating physiological metrics with landscape ecology; however, limitations in breadth and scope make conservation determinations difficult. For example, Janin *et al.* (2012) assessed hormonal (corticosterone) differences in adult and juvenile toads (*Bufo bufo*) moving through a landscape matrix (ploughed soil, meadow and forest) to assess whether corticosterone is indicative of landscape resistance to animal movement. Using lab- and field-based substrate choice experiments, the authors found that adult toads preferred forest litter over ploughed soil and had higher corticosterone levels when crossing ploughed soil, with no variation in corticosterone observed in juveniles. Overall, the study successfully integrated landscape ecology, behavioural ecology and physiology. However, the authors explained the differences between adult and juvenile corticosterone levels by making assumptions about the cognitive processing and sensory perception of the developmental stages of toads (i.e. the ‘black box’), without any direct data to support these assertions. Despite this ‘black box’, the authors attempt to scale to the population by discussing how corticosterone influences population dynamics, outlining specific effects including changes in amplexus behaviour, fertilization probability and adrenal desensitization. Avoiding such assumptions are key in this case, as elevated glucocorticoids are not universally a signal of stress and may be adaptively elevated to achieve specific life cycle needs (e.g. pre-migratory fattening; Holberton, 1999).

Macrophysiology, an approach where physiological markers are investigated over large spatial and/or temporal scales, may be an effective way to link variation in habitat quality (at the individual level) to impacts on populations or meta-populations (Chown and Gaston, 2008; Table 2). This approach differs somewhat from landscape physiology, which is primarily concerned with determining how changes in landscape impact physiology. Lea *et al*. (2018) successfully used macrophysiology and the integration of multiple physiological, ecological and demographic measurements to scale up to species-level impacts to investigate poor population growth rates of the vulnerable Cape Mountain Zebra (*Equus zebra zebra*). They demonstrated, through investigation of hormone profiles, that poor population growth rate was ultimately linked to poor habitat quality. Despite the obvious strengths of a macrophysiological approach in identifying large-scale stressors and promoting species-level conservation, this approach has primarily been used in assessing thermal tolerances in relation to global temperature change (Chown, 2012; Chown and Gaston, 2016). We suggest that macrophysiology could be used to identify physiological impacts of numerous other stressors including chemical pollution, habitat quality, urbanization and precipitation. For example, the use of non-invasive biomarkers (e.g. coloration of scales and feathers) as indicators of pollution exposure potentially allows for large-scale assessment of pollution impacts (Lifshitz and St Clair, 2016); however, using the bio-marker method must be done carefully to avoid becoming another ‘black box’, as these traits can vary for multiple reasons, including natural differences in individual quality. Researchers interested in meta-population or species-level conservation should maximize the spatial and/or temporal scope of their study by incorporating macrophysiology into their research, collaborating with researchers in disciplines that track larger-scale population trends (e.g. population ecologists), or participating in working groups that facilitate data collection at large scales (Table 2).

Anthropogenic environmental stressors are a pervasive threat to global biodiversity that can directly impact physiological processes and fitness. Examining physiological responses to these stressors, including rate of uptake, tolerance and toxicity, have been the basis for many population risk assessments and mitigation efforts and have informed regulatory measures aimed at conserving populations (Madliger *et al*., 2016; Peterson *et al*., 2017). However, assessments and mitigation efforts that rely on a ‘black box’ to link environmental stressors with physiological responses and fitness consequences may not effectively scale to the meta-population or species level as stressors vary across spatial and temporal gradients. One example that has significant potential in bridging this gap is the study by Hernout *et al*. (2018). They incorporated spatial patterns of metal concentrations in soil and prey items, as well as diet preferences and consumption rates, for several species of insectivorous birds to determine each species’ level of risk from metal contaminants in the soil. The authors also compared the variation in metal toxicity risk based on species-specific breeding range, migratory status, and distribution patterns. They found that resident species like the blackbird (*Turdus merula*), that have wide distributions overlapping urban areas and diets of ground-dwelling invertebrates, are expected to be at risk of metal toxicity over a large portion of their range, not just from specific areas with significantly elevated metal concentrations. A key next step to scaling up farther is to ground-truth predictions by examining bird tissue samples for heavy metals (but see Hernout *et al*., 2013), which would lead to a deeper understanding of how metal accumulation in birds varies with life history traits and biomagnification through trophic webs. Determining the degree to which species are at risk (i.e. experiencing sublethal, chronic or acute effects) from human-induced stressors requires tracking spatiotemporal and life stage variation in exposure (see Box 2).

### From populations and species to higher levels of the biological hierarchy

Because increasingly complex levels of the biological hierarchy display emergent properties that may not be apparent at simpler scales, it can be difficult to delineate the potential role of physiology in addressing large-scale conservation problems. Beyond the challenge of detecting physiological and behavioural responses in individuals and directly relating those responses to fitness and population declines, to effectively inform conservation we need to think about how those individual responses (e.g. a fish moving to deeper water to avoid warming trends (Box 1), or a bird leaving overwintering grounds late because of reduced precipitation (Box 2)) translate to changes in higher levels of the biological hierarchy. In Fig. 1, the layers of the landscape become increasingly complex as we move up the hierarchy, and we must, therefore, integrate the way individuals and populations interact with the abiotic and biotic environments. Here we explore ways that different research disciplines may provide insight at these larger scales.

### Interspecific interactions

Scaling beyond the population and species level of the biological hierarchy can be achieved by considering the role of physiology and behaviour in mediating interspecific interactions. In particular, work focused on understanding the role of competition between native and invasive species and resultant community shifts can elucidate links from individual physiology through community-level dynamics. Trait variation, both within and between populations, predicts an association between behaviour and environmental characteristics of populations in which certain behavioural traits are selected for in familiar, altered or new environments (Dingemanse *et al*., 2007). The variation brought about by physiological mechanisms can influence the community in which a population lives, especially via interspecific competition and predator-prey interaction. For example, the endangered Fijian ground frog (*Platymantis vitiana*) exhibits significantly reduced body condition, increased urinary corticosterone metabolites, suppressed sex steroid metabolites and reduced reproductive success due to competition with invasive cane toads (*Rhinella marina*; Narayan *et al*., 2015). Likewise, both non-western mosquitofish (*Gambusia affinis*) and American bullfrogs (*Lithobates catesbeianus*), invasive in California wetland communities, have reduced native amphibian growth rates and body mass due to a reduction in foraging time activity level (Preston *et al*., 2012). Ultimately, the invasive mosquitofish and bullfrogs have led to changes in native species abundance and occupancy resulting in overall shifts in community composition.

The ‘landscape of fear’ (LOF) is another potential indirect predator–prey interaction that could help scale to the community and ecosystem levels. Seminal LOF work has focused on the Yellowstone National Park ecosystem following the reintroduction of grey wolves (*Canis lupus*) from 1995 to 1996 (Smith *et al*., 2003). The reintroduction of wolves led to increased vigilance and shorter foraging time in ungulates, as well as reduced concentrations of the reproductive hormone progesterone, ultimately leading to lower ungulate population sizes (Creel *et al*., 2007; Laundré *et al*., 2010). In addition to direct physiological impactions, the LOF modified the movement and foraging behaviour of ungulates such that riparian plant communities were restored, culminating the ecosystem-level trophic cascade (Beschta and Ripple, 2016). In another example of the LOF influencing community and ecosystem function, mesocarnivore (i.e. raccoon, *Procyon lotor*) foraging of intertidal and subtidal prey was reduced following vocalization playbacks of large carnivore predators in the Gulf Islands of BC, Canada (Suraci *et al*., 2016). Reduced mesocarnivore predation leads to a drastic increase of intertidal raccoon prey including red rock crabs (*Cancer productus*), subsequently leading to a decrease in one of the crab’s competitors, staghorn sculpin (*Leptocottus armatus*) and one of their prey items, the periwinkle snail (*Littorina scutulata*). The evaluation of the LOF in this system revealed important ecosystem functions that may otherwise have been overlooked, and pursuing LOF studies could be a valuable link in scaling from the individual to community and ecosystem level.

The spatial distribution of risk is a key part of the LOF, but evaluating the temporal variation can also offer increased resolution of LOF effects. The LOF is dynamic over various time scales: across seasons, lunar cycles and even at a daily scale. For example, bright moonlight seems to increase predation risk in nocturnal rodents by increasing the ability of predators to detect prey and leads to decreased activity in rodents (Daly *et al*., 1992; Prugh and Golden, 2014). Decreased activity could lead to reduced foraging time, which may affect nutritional needs of rodents, potentially leading to demographic consequences (Zanette *et al.*, 2014). In the Yellowstone system, Kohl *et al.* (2018) found that predation risk to elk was temporally dynamic such that elk used risky habitats during periods in which wolves were less active. Shifting activity time may cause temporal mismatches between different forage items that may be key for physiological regulation. Further, many physiological responses vary over time, such as seasonal variation in corticosterone in snakes (Dauphin-Villemant and Xavier, 1987) and metabolic rate variations in response to diel hypoxia fluctuations in catfish (*Silurus meridionalis*) (Yang *et al.*, 2013). Understanding temporal variation in the LOF is key to capturing a complete understanding of how interspecific interactions influence communities and ecosystems.

### Landscape ecology

To scale population- and species-level data to higher levels of the biological hierarchy, it is necessary to determine the role of abiotic variation in driving animal physiology across the landscape (Fig. 1). Abiotic gradients (e.g. temperature, moisture, light, land use) exist across multiple scales, including latitude, elevations and small-scale topographic complexity (e.g. slope, aspect) and interact to drive variation in availability and quality of resources which will ultimately influence individual physiology including metabolism, water loss rates, and stress (McCain and Grytnes, 2010). Depending on the adaptive capacity of the population (i.e. the capacity for the population to respond plastically and/or genetically to environmental change), we may be able to predict population and species responses across a larger scale. The growing threat of HIREC has accelerated our need to understand species responses to change across the landscape, and physiological metrics such as stress provide a direct link to such understanding. Ultimately, such knowledge can be used to open the ‘black-box’ and scale to higher community and ecosystem levels. Further, leveraging abiotic gradients may provide useful ‘natural experiments’ towards predicting responses to HIREC and offer insight into physiological traits that may be successful in the face of change (Körner, 2007). Through exploiting natural, landscape-scale abiotic variation, Riddell and Sears (2015) provided novel insight into physiological trait variation and regulation of dehydration in lungless plethodontid salamanders under a suite of environmental conditions. Physiological variation as a function of abiotic conditions was further used to predict responses to future climate shifts, suggesting that salamanders may be more resilient to climate change given their observed local adaptations to warm and dry conditions (Riddell *et al*., 2018). Following up on this study by assessing other forest processes or organisms that are directly or indirectly impacted by salamanders could provide a unique opportunity to further scale these results up the biological hierarchy.

### Movement ecology

Many animals migrate to avoid seasonal extremes in climatic conditions; however, they are often still subjected to extreme variations in climate and land use across their migration paths (Box 2). Migratory life history strategies are positively correlated with higher metabolic rates and feeding rates (Metcalfe *et al*., 1995; Cutts *et al*., 1999; Lahti *et al*., 2002, Morinville and Rasmussen, 2006), possibly due to the need for sustained activity during migration (Biro and Stamps, 2010). For example, during salmonid migrations, populations experience extreme variation in temperature in both the horizontal and vertical dimensions of a stream, which influence many key physiological measures. Many river systems have been altered, either through human (e.g. dam construction or removal) or climate (e.g., flooding events, rising water temperature) impacts, causing shifts in both hydrological and thermal conditions. These altered environments create additional challenges for migrating salmon (Fenkes *et al.*, 2016). Increased temperatures and flow regimes lead to higher metabolic rates and cost of transport, and often result in decreased spawning success (Fenkes *et al*., 2016). The energy trade-off between migratory demands—which are largely driven by thermal and hydrological regimes experienced throughout the upstream migration route—and reproductive output can have cascading effects throughout an ecosystem, since salmon serve as keystone species driving many ecosystem processes (Schindler *et al*., 2013; Tonra *et al*., 2016). Without the investigation of dynamic abiotic gradients, a comprehensive understanding of responses to change is impossible. Perhaps paramount among research needs in this area is a greater understanding of the physiological mechanisms that underlie movement behaviour (e.g. migration; Jachowski and Singh, 2015). An emerging solution to this gap in knowledge for many species is advances in biologging/biotelemetry technology, allowing for simultaneous measurement of physiological parameters and geographic position (Donaldson *et al.*, 2014; Jachowski and Singh, 2015).

### Community ecology and ecosystems

Direct examples of connections between individual-level stress and community and ecosystem dynamics are key in the implementation of conservation and management schemes; however, such examples are rare and largely comprised of reviews synthesizing existing studies. For example, Zapata *et al*. (2019) provide a thorough review of a single environmental stressor (artificial lighting at night) and a specific ecosystem (estuaries) to make connections among published examples at each level of the hierarchy. They discuss the implications of light infiltration at scales relevant to individuals of various species (e.g. artificial lighting at night suppresses the expression of melatonin in some fishes, thereby promoting increased activity and foraging at night; Ouyang *et al*., 2018) and how this might scale to ecosystems through altered food web linkages. Zapata *et al*. (2019) suggest a suite of future research avenues, specific to light pollution and aquatic ecosystems, that could broadly apply to the studies highlighted here. For example, there is a need to characterize the stressor landscape at multiple scales, understand the physiological responses at those scales and then integrate disciplinary knowledge across the biological hierarchy to make science-based mitigation decisions.

## Key challenges and recommendations

While examples of successful conservation physiology studies integrated across disciplines are emerging, there are many obstacles that stand in the way of studies successfully scaling up the biological hierarchy. Here, we outline some of these key challenges and suggest potential solutions for researchers and managers trying to increase the scope of their studies. We do not expect that single studies will be able to scale all the way from physiological responses of individuals to ecosystem functioning, as this is an unrealistic expectation. However, collectively, through careful study design and collaboration, we can achieve the goal of scaling up the biological hierarchy. See Table 2 for additional tools that can be used to scale up research efforts.

- Lack of funding and personnel: scaling up often requires larger operating budgets and more personnel spread across numerous locations. Working groups or other collaborations (Table 2) can often be leveraged for funds or provide the opportunity for data collection across a greater area without incurring additional costs. In some cases, involving the public in citizen science data collection can also increase the scope of the study.
- Available time: university research is often carried out by graduate students and thus limited to 2–4 years to allow degree completion. Principal investigators can establish long-term studies, where individual graduate projects take steps to scale up the research, gradually building the dataset. Researchers can also design studies that take advantage of existing longitudinal datasets or previously well-studied systems where some mechanistic understanding already exists but has not been explicitly scaled up.
- Initial study design (i.e. choosing the ‘right’ metrics): researchers should place an emphasis on collecting metrics appropriate to their study system that can be used to scale up the hierarchy. Often this means collecting metrics from different disciplines that can then be used in conjunction. Currently, there is no consensus on which metrics are best for scaling up; however, focusing on metrics that have previously been successful in similar systems and newly emerging metrics may improve our ability to scale up.
- Disciplinary expertise: since scaling up requires both depth and breadth of knowledge, it is often necessary to perform research outside of one’s primary area of expertise. Given the extent and depth of literature within fields, we suggest that disciplinary breadth will best be achieved by collaborating with researchers across specializations. However, since communication can be difficult across disciplines (e.g. due to disciplinary jargon and different standards), it is important to communicate frequently with collaborators during every step of the project starting with initial study design.
- Taxa-specific and logistical challenges: Some research challenges are unique to particular study organisms or systems (e.g. endangered or rare species, difficult socio-political circumstances). Communication with researchers, managers, indigenous communities or local organizations with experience working in that area, or forming working groups to synthesize knowledge and increase group expertise, could help address these logistical challenges.
- Integration of theoretic approaches: Models used to scale up are often challenging to parameterize and require quantities of data beyond the scope of many studies, though may provide important predictive power in moving toward conservation and restoration goals. This again may be overcome by forming collaborations, such as working groups, or utilizing public access databases.

## Conclusions

Interdisciplinary research, that combines principles and techniques from multiple physiological (endocrine, nutritional, sensory) and ecological (landscape, behavioural, evolutionary) disciplines to scale the biological hierarchy, will ultimately be the most successful in addressing large-scale conservation threats currently facing biodiversity. While the need to integrate research across disciplines has been generally acknowledged, there is still much that can be done to more effectively inform conservation practices. Population-level effects are often assumed based on physiological principles, but these ‘black box’ assumptions are often missing mechanisms that would explicitly link levels of the biological hierarchy. Additionally, many population-level studies do not identify the mechanistic, physiological responses exhibited by the individual (though it is often assumed). For some conservation management, simply investigating one level of the biological hierarchy may be sufficient; however, in more complex systems that are experiencing increasing anthropogenic influences and multiple stressors, investigation across multiple levels, achieved through collaboration among disciplines (Cooke and O’Connor, 2010), may be the best method to increase the ability of managers to successfully conserve species (see Box 1).

Understanding the individual responses to stressors is key to predicting change; however, individual-level responses may not necessarily be reflected in population-level responses. For example, Madliger and Love (2016) measured glucocorticoid levels in female tree swallows (*Tachycineta bicolor*) to assess their correlation with body condition (i.e. experimentally manipulated foraging ability), habitat quality and fitness. While this study demonstrated that lower quality habitat and body condition can alter baseline glucocorticoids long-term, concomitant effects to fitness (e.g. offspring mass and reproductive output) were not observed. Thus, an understanding of individual responses does not necessarily give a full indication of the magnitude of the population-level response. In order to remove these ‘black boxes’, we must instead determine the intensity at which a response must occur in order to elicit population-level responses (Chown *et al.*, 2010). This scaling-up may reveal that negative individual impacts may not be severe enough to alter population levels; however, it may also reveal specific physiological processes that play a major driving role in population dynamics.

Species do not exist in isolation, so in order to fully understand how to conserve species at risk, we must also consider the diversity of landscapes they experience and how these varied landscapes are likely to influence the responses of organisms to anthropogenic change. We must include species across ranges, migratory paths or within watersheds in order to grasp the species and ecosystem-level responses to anthropogenic change. Using tools such as macrophysiology can inform us on these multiple scales to indicate how physiology is affecting these populations across these landscape features, thereby indicating critical areas in which conservation efforts can be targeted. Integrating physiological mechanisms across multiple levels of the biological hierarchy can seem like a far-fetched challenge for researchers and conservation managers. Through collaborative, interdisciplinary research, these goals can be attainable. While the push exists for integration of conservation physiology within individual fields, there is still a need to further increase our breadth of knowledge across multiple fields, which may illuminate what has otherwise previously only been a ‘black box’.

## Box 1: Opportunities for scaling up: integrating stickleback research across disciplines

In many cases, integration across disciplines and levels of the biological hierarchy to better inform conservation is not hindered by the desire of researchers, but rather the lack of comprehensive data and resources (see Key Challenges and Recommendations). Previous reviews have highlighted connections made among disciplines where possible (e.g. conservation of Pacific salmonids; reviewed in Cooke *et al.*, 2012). Here, we highlight one case study where collaboration across multiple disciplines could provide significant conservation insights. The threespine stickleback (*Gasterosteus aculeatus*) could be considered a poster child for a number of disciplines, including ethology, evolutionary biology, sensory ecology and speciation genomics. For example, the threespine stickleback is a model organism for evolutionary biology, used to understand the process of speciation across its wide geographic range (McKinnon and Rundle, 2002). While a large body of literature exists for threespine stickleback, there has been little synthesis on how this knowledge can inform conservation or the implications for ecosystem function (Taylor *et al.*, 2006). Synthesizing literature across discipline-specific studies can provide important information about responses to environmental change and offers a unique opportunity to scale up the biological hierarchy.


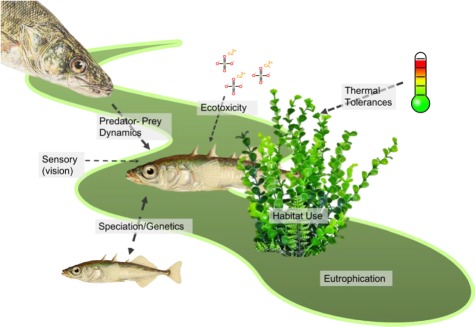


A study from sensory ecology found sticklebacks from populations native to lakes with differing photic regimes have distinct spectral sensitivities (i.e. individuals have cone photoreceptors in the retina maximally sensitive to different wavelengths of light; MacDonald and Hawryshyn, 1995), indicating that intraspecific variation in visual sensitivity may play a role in their ability to adapt to changes in the photic environment. Further, there is a direct link between variation in visual sensitivity and mate choice for male throat colour (Boughman, 2001). Thermal preferences have also been determined for sticklebacks, with thermal optima shifting to increased temperatures after acclimation (Garside *et al.*, 1977). The ability to thermally acclimate to increased temperatures may indicate an advantage for this species in the face of climate change. However, even though this species is known to be tolerant of warming, Moran *et al.* (2010) found that increases in temperatures of 4°C, when coupled with eutrophication, resulted in a complete loss of the stickleback population, which was not observed with increases in temperature alone. The specific physiological mechanism is unknown (i.e. a ‘black box’); however, much loss in biomass was attributed to severe hypoxic conditions (Moran *et al.*, 2010).

Integration across disciplines and scales within stickleback research has already begun. For example, Matthews *et al*. (2010) found that habitat use was correlated with trophic position, as sticklebacks in lakes with both benthic and limnetic populations held different trophic positions. Continuing to bridge disciplines at multiple scales can allow for increased insight into how this species will be able to cope with rapid environmental change. For example, there is evidence that increased eutrophication weakens sexual selection through increasing time and energy spent on courtship, while simultaneously weakening the strength of selection on red coloration (Candolin *et al.*, 2007). This study successfully addresses how individuals may alter behaviours in the face of environmental change. Continuing this work to uncover the physiological mechanisms by which these changes occur and direct population-level effects, such as avoidance of algal turbidity or changes to population biomass, offers an opportunity to scale up (Candolin 2019; Olsson *et al.*, 2019). Scaling this research to community and population levels can provide insight into long-term effects of eutrophication (and other concomitant stressors such as elevated temperature and hypoxia). Mechanistic modelling is one area in which individual-level processes such as physiology can be scaled up to inform population dynamics (Johnston *et al.*, 2019). The mass of data on stickleback responses to both natural and human-induced, rapid environmental change provides a useful example of both breadth and depth in understanding that has the potential to be scaled to higher levels by bridging disciplines. Systems like this can serve as starting points to unravelling the intricacies of species-environment interactions and help us to begin to break down the ‘black box’ in order to increase conservation action.

## Box 2: Integrating conservation physiology across the full annual life cycle to inform conservation

For migratory organisms, successful conservation strategies must address threats occurring in disparate locations across the full annual cycle of the organism to achieve maximum potential for success (Marra *et al*., 2015a). In North America, many climate change vulnerability assessments and plans fail to consider the migratory status, migratory connectivity, or non-breeding locations of imperilled species (Small-Lorenz *et al*., 2013; but see Culp *et al*., 2017). This is not surprising, as conducting research across annual cycle stages in migrants, and applying these links to population-level conservation, can be challenging, especially for small, difficult-to-track species. Many studies have employed emerging techniques with both intrinsic (e.g. stable isotopes; Tonra *et al*., 2011) and extrinsic (e.g. light-level geolocators; Tonra *et al*., 2019) markers to overcome limitations in spatial data. Integrating conservation physiology with these approaches provides an important tool for understanding the underlying mechanisms that limit fitness both within and between seasons. A valuable example of integrating physiology into the study of full annual cycles of migrants is the American Redstart (*Setophaga ruticilla*; hereafter redstarts) study system.

The redstart system is instructive in the extent to which different portions of the annual cycle can be linked, as research on this system has identified both individual-level carryover effects and population-level seasonal interactions. On the redstart wintering grounds, research in Jamaica has established links between habitat moisture, availability of food resources and body condition (e.g. Studds and Marra, 2011), physiological stress (baseline and stress response corticosterone levels; Marra *et al*., 1998) and migration phenology (e.g. Studds and Marra, 2005). Importantly, through experimentation, researchers have demonstrated causal links between habitat, food availability, body condition and behaviour. Cooper *et al.* (2015) experimentally reduced food availability and showed adaptive fat regulation and a trade-off with muscle mass, which subsequently affected migratory readiness and departure from the winter grounds. These studies powerfully demonstrate the effects of habitat on energetics, which are operating independent of intrinsic differences in quality among individuals.

Within the redstart system, physiological impacts of moisture gradients generate sub-lethal carryover effects onto breeding productivity. Individuals from wetter winter habitats arrive earlier to breeding sites, and produce more offspring, than those from drier habitats (Marra *et al*., 1998; Norris *et al*., 2004). In males, testosterone (T) appears to drive this relationship as individuals begin physiological breeding preparation on the wintering grounds (Tonra *et al*., 2013). Body condition is positively related to pre-migratory levels of T on the wintering grounds, and experimental elevation of T induced advanced migratory departure, through positive impacts on foraging rate, flight muscle and fat stores (Tonra *et al*., 2013). Males arriving to breeding sites early from wet winter habitats have higher circulating levels of T and are more likely to successfully breed (Tonra *et al*., 2011). Collectively, these studies demonstrate the numerous ways in which physiological mechanisms operating within individuals link disparate portions of the annual cycle, despite spatial and temporal separation.

While the redstart system has demonstrated the links between stages of the annual cycle at the individual level, conservation is primarily concerned with populations. Thus, it is critical to understand how these environmentally driven physiological mechanisms scale up to influence the growth rates of population segments. For instance, Marra *et al*. (2015b) evaluated 14 years of data on redstarts in Jamaica, demonstrating habitat-dependent population regulation, with density-dependent impacts on survival mediated through crowding effects on body condition. Scaling up further to the entire population, Wilson *et al*. (2011) found that plant productivity in the Caribbean in winter was a powerful predictor of population trends in the breeding range. Redstart abundance increased following wetter/more productive and decreased following drier/less productive, Caribbean winters. On the other hand, breeding ground plant productivity was unrelated to population growth. This system, with research at multiple levels of the biological hierarchy demonstrates that failing to consider conditions outside of breeding could obscure important processes driving population growth.

The redstart system provides a powerful example of how in-depth understanding of environmental drivers of physiological state can be used to understand interactions between seasons and population processes at larger scales. However, this level of understanding of a migratory organism, as with the stickleback example (Box 1), is the exception not the rule. It remains unclear how widespread carryover effects and population-level seasonal interactions are, though they have been described in a few other select systems (e.g. black-tailed godwit *Limosa limosa*; Gunnarsson *et al*., 2006). It is especially critical that such phenomena be understood for imperilled species, and if these relationships among life cycle stages exist, conservation action may be misdirected, if focused on a single stage of the annual cycle.
